# Quantification of Spectral Perception of Plants with Light Absorption of Photoreceptors

**DOI:** 10.3390/plants9050556

**Published:** 2020-04-27

**Authors:** Woo Hyun Kang, Jaewoo Kim, Hyo In Yoon, Jung Eek Son

**Affiliations:** Department of Plant Science and Research Institute of Agriculture and Life Sciences, Seoul National University, Seoul 08826, Korea; flatengine@hanmail.net (W.H.K.); plmokn78@snu.ac.kr (J.K.); yoonhi@snu.ac.kr (H.I.Y.)

**Keywords:** cryptochrome, incident spectra, light-emitting diode, light quality, photomorphogenesis, phototropin, phytochrome, plant factory

## Abstract

Although plant responses to artificial lighting spectra often produce abnormal morphogenesis and reduced productivity, no quantification method to determine how plants perceive and respond to light has been available. Our objective in this study was to test whether a plant’s spectral perception can be quantified using the light absorption of its major photoreceptors, phytochrome, cryptochrome, and phototropin. We developed an artificial solar lamp and three different light sources, based on a high-pressure sodium lamp, a fluorescent lamp, and red and blue light-emitting diodes, whose absorption by photoreceptors was equal to that of the standard solar spectrum. Cucumber plants grown under the artificial solar and developed light sources showed normal photomorphogenesis and were indistinguishable from each other. Plants grown under unmodified commercial light sources had abnormal photomorphogenesis that made them short and small. The photosynthetic rate was higher under the unmodified light sources; however, dry masses were highest under the artificial solar and modified light sources, indicating that the cucumber plants are optimized to the solar spectrum. Our results clearly demonstrate that the spectral perceptions of plants can be quantified using the light absorption of their photoreceptors, not visual color or spectra. We expect that our findings will contribute to a better understanding of plant perceptions of and responses to light quality, and improve the productivity of plants cultivated under artificial light.

## 1. Introduction

Light is an essential element of plant survival because it provides both the energy required for growth and information about the surrounding environment. To accommodate changing environmental conditions, plants sense light quality by absorbing light through their photoreceptors and then responding to the incident light quality. Generally, light quality is quantified using 100 nm color segments: ultraviolet (UV; 300–400 nm), blue (B; 400–500 nm), green (G; 500–600 nm), red (R; 600–700 nm), and far-red (FR; 700–800 nm). Plants have photoreceptors for each waveband: phytochrome for R and FR, cryptochrome and phytochrome for B and UV-A, and UVR8 for UV-B [1,2,3]. A plant’s response to each waveband accommodates the environmental condition it implies, thereby providing competitive advantages [4]. For instance, a plant’s response to blue light, which is common in the high irradiance of natural light, involves stomatal opening [5], thick leaves [6], and antioxidant accumulation [7]. High fractions of green and far-red light induce shade-avoidance syndrome, represented by an elongated hypocotyl and petioles to enable the plant to move out of shade [8,9] because those conditions occur when the red and blue light within solar radiation is filtered out by dense foliage above or nearby [10,11,12]. 

Using those understandings about plant responses to light quality, artificial lighting is widely used in research and commercial production to supplement or replace sunlight for better control of the physiological responses that are being researched or for better productivity and product quality [6,13,14,15,16]. However, the distinct light qualities of artificial lighting often produce undesirable responses in plants. For instance, intracanopy R and B light-emitting diode (LED) lighting on cucumber plants induced severe leaf curling and reductions in stem length that reduced light capture [17], whereas cucumber plants grown under a fluorescent lamp (FL) or high-pressure sodium (HPS) lamp showed substantially lower dry mass than those grown under an artificial solar (AS) spectrum due to shorter petioles and hypocotyls [6]. As these cases indicate, plants’ overall responses to specific light sources are not yet well understood [13,18].

Arguably, the current inability to predict a plant’s response to a given spectrum is caused by the inadequacy of using the current, color-segment method of understanding light to quantify what plants perceive. Plants respond to what they perceive, so a plant’s response to light should be predictable based on its perceptions. From this perspective, phytochrome photoequilibria (PPE) represent light quality using the state of two isoforms of the photoreceptor phytochrome [19,20,21]. PPE has shown linear relationships with the responses of various plant species [20,22,23]. However, PPE takes only phytochrome into account, omitting the other major photoreceptors such as cryptochrome and phototropin. Considering their significant role in plant responses to light quality and the intense interactions among all the photoreceptors in downstream signal transduction [2,24,25], the information about a plant’s spectral perception contained in PPE cannot fully represent a plant’s spectral perception. Thus, it remains difficult to answer some basic questions that many researchers and growers have encountered: What is the best way to predict a plant’s overall response to a given light quality, and what is the best way to blend different light sources to achieve specific goals [13]?

We hypothesized that a plant’s spectral perception can be fully quantified using the light absorption of the major plant photoreceptors (*σPR*) under any given light quality and light source, provided that *σPR* accurately reflects how plants perceive light quality. Our objective in this study was to test that hypothesis: to develop a method to calculate the light absorption of the major plant photoreceptors, phytochrome, cryptochrome, and phototropin, and to develop light sources based on those calculations and compare the responses of cucumber plants. 

## 2. Materials and Methods

### 2.1. Plant Material and Cultivation Conditions

Cucumber plants (*Cucumis sativus* L. ‘Jo-eun baekdadagi’) were sown in water-soaked urethane cubes (2 × 2 × 2 cm), germinated under darkness for three days, and then moved to 200 μmol m^−2^ s^−1^ of cool white fluorescent lamps (FL; Dulux 55W, Osram, Munchen, Germany) in a plant factory module. After 14 days, when the first true leaves were just appearing, the seedlings were transplanted to a noncirculating hydroponic system, with three plants per container with 5 L of Hoagland’s solution (pH 5.7–6.1; EC 1.8 mS cm^−1^). Nutrient solutions were supplemented when necessary. The photoperiod, day/night temperatures, relative humidity, and CO_2_ concentration were 16 h, 25 °C/15 °C, 50%, and 1000 μmol mol^−1^, respectively. The plants were subjected to 200 ± 10 μmol m^−2^ s^−1^ of photosynthetic photon flux density (PPFD) and harvested 21 days after transplanting (DAT). 

### 2.2. Spectral Data of Photoreceptors and Light Sources

**Figure 1:**
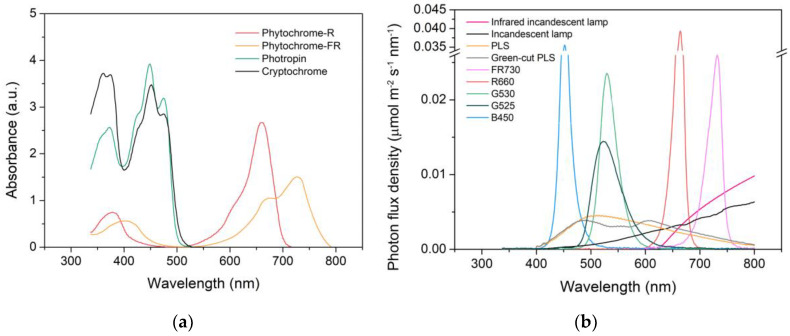
Spectral absorbance of plant photoreceptors (**a**) and emission spectra (**b**) of light sources used in this study. Spectral absorbances of photoreceptors were obtained from the literature (see Section 2.2). See Section 2.5 for abbreviations. a.u. indicates arbitrary unit.

The spectral absorbances of the major photoreceptors were obtained from the literature as follows: R-absorbing and FR-absorbing forms of phytochrome (*P*_r_ and *P*_fr_) [21], cryptochrome [26], and phototropin [27]. The emission spectra of the light sources used in the experiment were measured with a portable spectrometer (Blue-wave, StellarNet, Tampa, FL, USA). The emission spectra obtained from the light sources and the standard solar spectrum (G173-03) [28] were normalized by attenuating their amplitude until it had 1.000 μmol m^−2^ s^−1^ of PPFD within the 400 to 700 nm range. The obtained spectral absorbances of the photoreceptors were normalized by attenuating their amplitude until their light absorption under the normalized standard solar spectrum in the wavelength range of 400 to 800 nm, calculated with the following Equation (1), became specific values. For cryptochrome and phototropin, it was 0.500. For phytochromes, it was 1.000 for the sum of the values of two phytochromes, or total phytochrome (*P*_total_), in order to maintain the amplitude ratio between *P*_fr_ and *P*_r_.

(1) $$A=\overset{800}{\underset{400}{\sum }}N_{\lambda }\alpha_{\lambda }$$

where *A* is the amount of light absorption of a photoreceptor; *N_λ_* is the photon flux density at wavelength λ; and α_λ_ is the absorbance of the photoreceptor at wavelength λ. All data were prepared and calculated in 1 nm steps. If the obtained data had larger steps, linear interpolation was applied. Spectral data are shown in Figure 1.

### 2.3. Calculating the Light Absorption of Photoreceptors

The amount of light absorbed by the photoreceptors under each light treatment was calculated by summing the product of the normalized spectral absorbance of the photoreceptors and the normalized emission spectra of the light sources across the 400 to 800 nm range in 1 nm steps using Equation (1).

### 2.4. Light Quality Treatments

**Figure 2:**
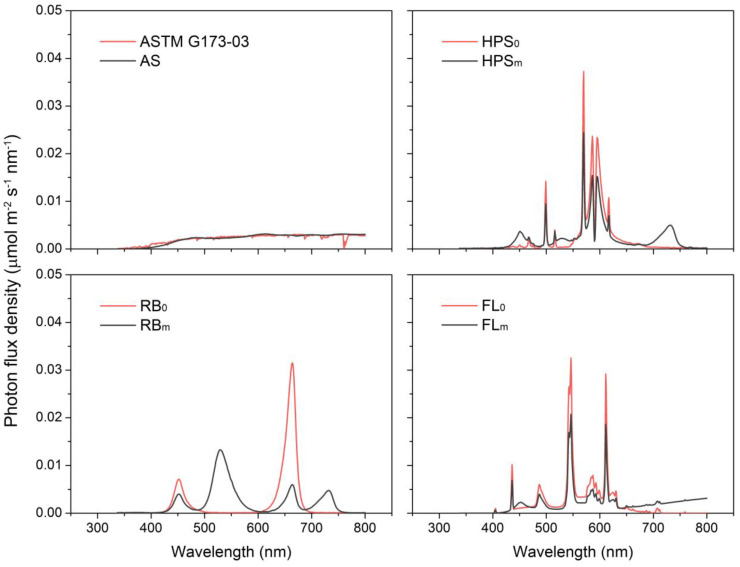
Emission spectra of light sources for each treatment condition. See Section 2.5 for specifics about the modified light sources.

We used seven spectral treatments and grew six plants under each treatment (Figure 2). An AS lamp, whose emission spectrum resembles the standard solar spectrum, was developed and used as a control. Three treatments were unmodified spectra under which cucumber plants were grown with conventional light sources: HPS_0_ (HPS SON-T 400W, Philips, Eindhoven, The Netherlands), RB_0_ (660 nm R (R660) and 450 nm B LEDs (B450, Hephas, Seoul, Korea), and FL_0_ (Dulux 55W, Osram, Munchen, Germany). The other three treatments were modified spectra in which the light qualities of growth irradiance from the abovementioned light sources were modified by substituting irradiance from other light sources to produce light absorption by each of the major photoreceptors equal to that under the standard solar spectrum: HPS_m_, RB_m_, and FL_m_. Figure 2 shows the spectra for each treatment.

### 2.5. Development of Light Sources

Four light sources, AS, HPS_m_, RB_m_, and FL_m_, were developed for this experiment. Table S1 provides the irradiance portions of the light sources in each treatment. AS was developed by a method modified from Hogewoning et al. (2010), using a sulfur plasma lamp (PLS; PLS 700W, LG Electronics, Seoul, Korea), a green-cut filter (Filter 1581, Gamcolor, Los Angeles, CA, USA), and incandescent lamps (200W Jangsoo Lamp, Seoul, Korea). Modification of the conventional light sources was carried out based on the calculated light absorptions of the photoreceptors, compensating for deficient or excessive values with additional light sources. Irradiance was maintained by reducing the irradiance of the original light sources. For HPS_m_, irradiance was replaced with 530 nm G (G530; Hephas, Seoul, Korea), B450, and 730 nm FR (FR730; JDR-IR730-50W, Jiaderui, Shenzhen, China) LEDs. In RB_m_, light from 525 nm G LEDs (G525; JDR-G525-100W, Shenzhen, China) and FR730 was added. In FL_m_, infrared-incandescent lamps and B450 replaced irradiance from FL_0_. Diffuse glass (DAGlass 301, D.A.Glass, Rzeszów, Poland) was installed between the light sources and the cucumber plants in all treatments to ensure optical equivalence among treatments. Walls in the growth room were covered with black fabric to minimize the reflection of the lights and consequent cross-contamination of light quality between treatments. 

### 2.6. Measurement of Growth Parameters and Morphological Characteristics

At the end of the photoperiod on DAT 28, the cucumber plants were photographed with a digital camera (EOS 600D, Canon, Tokyo, Japan) and a standard-angle lens (EX DC HSM 30mm f/1.4, Sigma, Kawasaki, Japan) to analyze their morphological characteristics with an image analysis software (ImageJ v1.49, NIH, Bethesda, MD, USA). Exposure values were fixed in all photos (f/4.0, 1/80s, ISO 400) with white-LED ceiling lamps as the sole lighting. To calculate the projected leaf area, each cucumber plant was photographed separately from above with reference scales placed at the same height as the upper leaves. The projected area ratio was calculated by dividing the projected leaf area with the total leaf area. Then, the plants were dissected into leaves, petioles, main stem, and root and laid on the floor before being photographed again. After photos were taken, the plants were oven dried at 80 °C for 72 h, and then their dry masses were measured with a precision scale. 

### 2.7. Measurement of Photosynthetic Rate

The in situ leaf photosynthetic rate under each treatment was measured with a portable photosynthesis meter (LI-6400, LI-COR, Lincoln, NE, USA) and a clear-top measuring head during the photoperiod on DAT 27. CO_2_ concentration, flow rate, and block temperature were 400 μmol mol^−1^, 500 μmol s^−1^, and 25 °C, respectively.

### 2.8. Statistical Analysis

Normality of the data was checked with the Shapiro–Wilk normality test; when this assumption was rejected, log transformation was applied. Fisher’s least significant difference test was used to make multiple post hoc comparisons among spectral treatment means from significant one-way analysis of variance tests using OriginPro 8 software (OriginLab Corp, Northampton, MA, USA).

## 3. Results

### 3.1. Light Absorption of Photoreceptors under Light Sources

The calculated light absorptions of the R- and FR-absorbing forms of phytochrome, cryptochrome, and phototropin (*σP*_r_, *σP*_fr_, *σCry*, and *σPhot*, respectively) under the spectral treatments are presented in Table 1, and the absorptions under all the light sources we tested are presented in Table 2. The emission spectra of the unmodified light sources, HPS_0_, FL_0_, and RB_0_, were deficient in FR compared to R, which produced a relative deficiency in *σP*_fr_ that constituted interestingly similar *σP*_r_/*σP*_total_ values of 0.733, 0.735, and 0.718, respectively (Table 1). Lack of FR and consequent *σP*_r_/*σP*_total_ values between 0.690 and 0.748 can be found in red, green, and white LEDs among the light sources we examined (Table 2). The blue photoreceptors, *σCry* and *σPhot*, exhibited small differences between them under most of the light sources, which is understandable given their mostly overlapping spectral absorbance (Figure 1a). 

**Figure 3:**
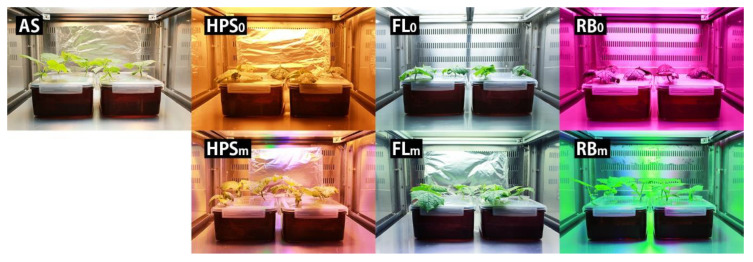
Visual appearance of each treatment and cucumber plants at 7 days after transplanting. See Section 2.4 for treatments.

Because all the modified light sources used in this study were supplemented with a considerable amount of additional FR, it is worth noting that the amount of additional FR required to produce an equal amount of *σP*_fr_ differed with the FR light sources because their spectral distributions were different (Figure 1b and Table S1). For instance, the *σP*_fr_ of FR730, whose emission spectrum is concentrated near the absorption peak of *P*_fr_, was 1.6 times higher than that of an infrared-incandescent lamp. Generally, LEDs showed either exceptionally high or low *σPR* values compared with other light sources as their monochromatic emission peaks overlapped with the absorption maxima or minima of the photoreceptors, respectively (Table 1 and Table 2). The *σP*_r_ of R660 was 3.8 and 5.2 times higher than those of HPS and FL, respectively, whereas that of G525 was 3.7 and 2.7 times lower, respectively. Because of this, relatively small differences in emission peaks could produce considerably different *σPR* values. For instance, the *σP*_r_ of R660 was twice as high as that of R630 (Table 2). As a result, the *σP*_r_ and *σCry* of RB_0_ were 3.5 and 1.3 times higher than those of the standard solar spectrum, respectively, at equal irradiance. To compensate for this excessive *σPR* in the RB_m_ treatment, 68.4% of the irradiance of RB_0_ had to be replaced with light from G525 (Table S1). On the other hand, because of the exceptional *σPR* values with the LEDs, the *σPR* adjustment of HPS_m_ and FL_m_ could be done with small amounts of substitution using B and G LEDs. Therefore, their spectra and visual color after the modification differed only slightly from before (Figure 2 and Figure 3). White LEDs showed rather moderate *σPR* values that were comparable to those under AS, HPS, and FL, including the deficiency in FR emissions (Table 1 and Table 2). It is notable that specific *σPR* values can be achieved using different light sources, colors, and spectra.

### 3.2. Photomorphogenesis of Cucumber Plants

Cucumber plants grown under the modified spectral treatments, HPS_m_, FL_m_, and RB_m_, showed photomorphogenesis equal to that of AS-grown cucumber plants and each other, fully expanded horizontal leaves, and long petioles and internodes, despite growing under substantially different spectra (Table 3 and Figure 2 and Figure 3). The total leaf area, number of leaves, petiole and internode lengths, and projected area ratio did not differ significantly among them.

**Figure 4:**
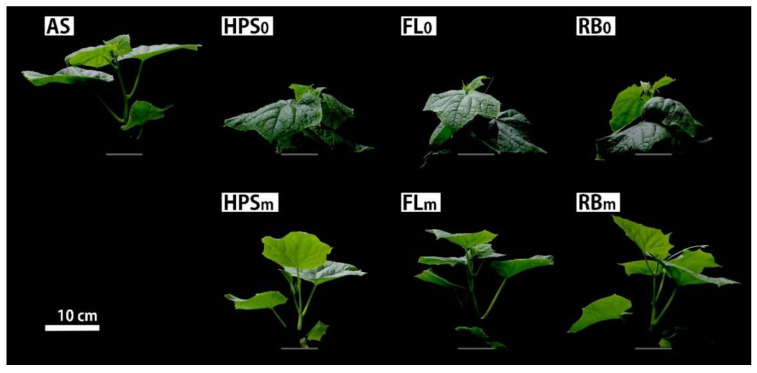
Photomorphogenesis of representative cucumber plants in each treatment at 21 days after transplanting. Grey bars indicate the ground level. See Section 2.4 for treatments.

On the other hand, the plants grown under the unmodified spectral treatments, HPS_0_, FL_0_, and RB_0_, had shorter and smaller plant architectures that differed conspicuously from those of the AS-grown cucumbers. The petiole length was 2.0, 2.1, and 2.0 times shorter in the plants grown under HPS_0_, FL_0_, and RB_0_, respectively, than in those grown under AS, and the internode lengths were 2.6, 2.0, and 2.1 times shorter, respectively. The ratio between the projected area and total leaf area was significantly lower under the unmodified spectral treatments than the modified spectral treatments. The projected area was 78.3% in the AS-grown plants and 77.8%, 79.2%, and 71.7% in the HPS_m_, FL_m_, and RB_m_ plants, respectively, without significant difference among them. On the other hand, it was 57.9% and 57.6% of the total leaf area under HPS_0_ and FL_0_, respectively (Table 3), whereas it was 43.6% under RB_0_, which was significantly smaller than the HPS_0_ and FL_0_ plants. In other words, compared with the AS condition, the plants grown under HPS_0_, FL_0_, and RB_0_ had a smaller portion of total leaf area exposed to the light because their photomorphogenesis was unfavorable for light capture. Furthermore, even though the number of leaves, total leaf area, and petiole and internode lengths did not differ significantly, the projected area ratio under RB_0_ was significantly lower than under HPS_0_ and FL_0_, because of severe leaf inclination and curling of the RB_0_-grown plants, as shown in Figure 4. 

### 3.3. Leaf Photosynthetic Rate and Plant Dry Mass

Contrary to the trend observed in the morphological traits, the in situ net photosynthetic rate (P_net_) was greater under the unmodified spectral treatments than under AS, in the order of RB_0_, FL_0_, and HPS_0_ (Table 4). The P_net_ of AS was slightly but significantly higher than those under the modified spectra, which did not show significant differences among themselves. Furthermore, the trends observed in the morphological traits were also found in the dry masses of the cucumber plants (Table 4). The total dry mass of the cucumber plants did not differ significantly between the cucumber plants grown under AS and the modified spectra, but it was 1.4, 1.5, and 1.3 times greater than those grown under HPS_0_, FL_0_, and RB_0_, respectively, although the difference was not significant in FL_m_.

## 4. Discussion

### 4.1. Photomorphogenesis of Cucumber Plants

Despite the overt differences in visual color and spectra of the modified light sources, equal light absorption by the photoreceptors produced equal photomorphogenesis in cucumber plants. This result clearly demonstrates that a plant’s response to light quality is governed by the light absorption of its photoreceptors rather than visual color or spectra. Thus, we have demonstrated that light sources can be mixed quantitatively to obtain specific photomorphogenic characteristics, and the overall photomorphogenic response of a plant to a given light quality can be predicted using *σPR*. It was petiole and internode lengths that caused the conspicuous differences in plant architecture found between the cucumber plants grown under modified and unmodified spectra. Under the modified spectral treatments, the addition of FR apparently caused an elongation of the petioles and internodes that made their photomorphogenesis comparable to that of plants grown under AS. Unlike previous studies in which the amount of additional FR was determined arbitrarily, we quantitatively calculated the amount of supplemental FR required by different light sources to induce photomorphogenesis of the cucumber plants grown under them indistinguishable from that of plants grown under AS. Under the unmodified spectral treatments, substantially shorter internode and petioles prevented the proper arrangement of leaves, making the overall plant architecture smaller than it was found under the modified spectral treatments. We did not expect their internode and petiole lengths to be indistinguishable, considering the differences in light quality among the modified light sources. Apparently, that occurred because the lack of FR in the three unmodified spectra matched their *σP*_r_/*σP*_total_ values. Given that the elongation of petiole and internode lengths are regulated by phytochrome and cryptochrome in an antagonistic manner [10,29,30,31], the influence of phytochrome apparent in our result is understandable. However, cryptochrome did not seem to have a noticeable effect on the photomorphogenesis of cucumber plants. Although *σCry* was 2.3 and 3.8 times higher under FL_0_ and RB_0_, respectively, than under HPS_0_, the cucumber plants grown under those three conditions did not differ significantly in their petiole and internode lengths. Also, the 25.3% lower *σCry* value under AS compared with RB_m_, which happened because AS was made to spectrally resemble the solar spectrum, did not produce a difference in those plants either. Those results suggest that phytochrome’s influence was greater than that of the blue photoreceptors, that the inhibition of elongation by the blue photoreceptors was saturated, or that low sensitivity to B light is a species- or cultivar-specific characteristic [32]. 

The plants grown under RB_0_ appeared smaller than those grown under HPS_0_ and FL_0_, which seems to be related to the exceptionally high *σP*_r_ and *σP*_fr_ values under the RB_0_ spectrum and could indicate another influence of phytochrome on the photomorphogenesis of cucumber plants. The *σP*_r_ and *σP*_fr_ values under RB_0_ were 3.0 to 4.5 times higher than those under HPS_0_ and FL_0_ because of the exceptionally high *σP*_r_ and *σP*_fr_ produced by the R LED. Considering that plants sense irradiance through phytochrome [33], the RB_0_ spectrum’s high *σP*_r_ and *σP*_fr_ could have been perceived as high irradiance, which would suggest that the observed leaf inclination and curling was a response to high light conditions intended to reduce light capture. A similar result was reported that severe leaf curling of cucumber plants occurred under R and B LEDs, unlike those grown under HPS, mentioning that it seemed like a light-avoidance response even though the irradiance was far from saturation [17]. The fact that perceived irradiance produced only different leaf inclination and curling, while the total leaf area, leaf number, and petiole and internode lengths remained unchanged, suggests that the different light parameters that plants perceive regulate different photomorphogenic features, which means that it might be possible to control individual photomorphogenic features by the precise manipulation of light.

### 4.2. Productivity of Cucumber Plants under Artificial Lighting 

As for the productivity of the plants, it is interesting that the lower P_net_ of the cucumber plants grown under the AS and modified spectral treatments, HPS_m_, FL_m_, and RB_m_, produced greater dry masses than the higher P_net_ found in the plants grown under the unmodified spectra. Increases in number of leaves and leaf area are associated with increased dry mass gain caused by increased light capture, thereby increasing the productivity of the plant [34,35]. However, differences in number of leaves and leaf area were not significant among treatments. Apparently, the large and expanded photomorphogenesis of the plants grown under the AS and modified spectra, which is advantageous for light capture, was behind this result: the accumulated assimilation was greater because the difference in light capture caused by the different photomorphogenesis exceeded the difference in P_net_. This result is in line with previous studies that cultivated cucumber plants under AS, HPS, and FL [6] or PLS and a metal halide lamp [36]. It is also consistent with previous reports that adding FR irradiance caused greater accumulated assimilation because the resulting larger plant morphology offered better light capture [23,37]. Although the abnormally short size of plants grown under artificial lighting and consequently lowered productivity have long been known [17,36,38], as well as the possibility of increasing productivity by adding FR light, modifying the light quality of artificial lighting is uncommon [6]. Our results suggest that light quality modification is necessary for artificial lighting, particularly that using R and B LEDs. However, the overall long-term outcomes of such modifications should be evaluated in future work.

Although the leaf level photosynthetic rate is often used as a parameter of plant productivity, it does not necessarily represent the dry matter production because many factors lie between leaf level photosynthesis and whole plant assimilation [35,39]. Our results show that the influence of light capture on accumulated assimilation can outweigh that of P_net_. In this study, the highest P_net_ was found in the RB_0_-grown plants, but it was overridden by the lower light capture that resulted from the plants’ small size, so they ended with a lower dry mass than plants with significantly lower P_net_ grown under other spectra. Our results also indicate that R and B LEDs might provide inappropriate light quality for plant cultivation. R and B LEDs are used for plant cultivation because plant pigments absorb their light efficiently, and plants grown under them have high leaf-level P_net_ [13]. However, as shown by the plants grown under RB_0_, its exceptionally high *σPR* values combined with the intense irradiance required for plant cultivation can be disadvantageous for plant productivity, outweighing the gain from high leaf-level P_net_. Also, high leaf-level P_net_ does not necessarily result in high plant-level P_net_ because high absorbance in leaves can deteriorate the light distribution within the leaf and canopies [40,41]. Therefore, the productivity of a specific light quality should be assessed not only by P_net_, but also using long-term and larger-level effects such as light capture and canopy photosynthesis [39]. 

The higher productivity of cucumber plants under AS and its equivalent spectral treatments (HPS_m_, FL_m_, and RB_m_) compared with the unmodified light sources, indicates that plant photomorphogenesis is optimized to the solar spectrum in terms of productivity, as would be evolutionarily expected, although different cultivation conditions and purposes could set different optima that would necessitate a certain degree of fine tuning in the light quality. For instance, size optimization for a given density and differences in plant-level and canopy-level optima could necessitate adjustments in the light quality. Also, our results indicate that artificial lighting spectra are not intrinsically different from the solar spectrum in terms of plant perceptions and responses. 

### 4.3. Light Absorption of Photoreceptors and Color Segments

When comparing the parameters of *σPR* and color segments, it is notable that significantly different *σPR* values can be produced by equal color values. For instance, light from R660 and R630 is in the same color segment; however, their *σP*_r_ values differ 1.9-fold. Likewise, *σP*_fr_ values of FR730 and infrared-incandescent lamps differ 1.6-fold. Considering the common use of red LEDs in plant experiments, this finding is concerning because it implies a possible failure in the control of variables. Thus, we argue that it is appropriate to use *σPR* as a variable in experiments rather than parameters from color segments such as R:FR or R:B. In addition, it is also concerning that “white” is often used as an experimental variable in the literature, along with color segments. It is because an unlimited set of spectra can be “white” since “white” is defined using human vision; furthermore, that definition is independent of and therefore irrelevant to the spectral perception of plants, as shown in this study. For instance, as presented in Table 2, several light sources that can be considered “white,” namely FL, white LEDs, and solar radiation, exhibit considerably different *σPR*. Thus, although the term “white” implies a spectrum equivalent to solar radiation or an imaginary light source with homogeneous spectra across the range, it does not actually define a specific light quality in any way, and the use of “white” as an experimental variable could thus cause a failure in the control of variables. 

## 5. Conclusions

In this study, we reproduced the response of cucumber plants to the solar spectrum using three different artificial lighting spectra to demonstrate that plants’ spectral perception can be quantified using photoreceptors, rather than visible color or spectra. Our results indicate that artificial lighting could be made more productive by modifying and tuning the spectra and that σ*PR* is an appropriate experimental variable for light-quality studies in plants. We expect that our findings will contribute to a new understanding of plant perceptions of and responses to light quality, and improve the productivity of plants cultivated under artificial light.

## Figures and Tables

**Table 1:** Calculated absorptions of plant photoreceptors per 1 μmol m^−2^ s^−1^ of photosynthetic photon flux density for the different spectral treatments (see Section 2.4).

Treatment	Absorption of Photoreceptors (Arbitrary Unit)				
	*σCry* ^1^	*σPhot* ^2^	*σP*_r_^3^	*σP*_fr_^4^	*σP*_r_/*σP*_total_^5^
AS	0.423	0.416	0.520	0.484	0.518
HPS_0_	0.173	0.154	0.604	0.220	0.733
FL_0_	0.397	0.401	0.440	0.159	0.735
RB_0_	0.652	0.606	1.835	0.719	0.718
HPS_m_	0.509	0.507	0.536	0.476	0.530
FL_m_	0.525	0.514	0.527	0.471	0.528
RB_m_	0.566	0.580	0.567	0.493	0.535

^1^ Light absorption of cryptochrome; ^2^ Light absorption of phototropin; ^3^ Light absorption of red-absorbing form of phytochrome; ^4^ Light absorption of far-red-absorbing form of phytochrome; ^5^ Absorption ratio between red-absorbing form of phytochrome and total phytochromes.

**Table 2:** Calculated absorptions of plant photoreceptors per 1 μmol m^−2^ s^−1^ of photosynthetic photon flux density for different light sources.

Light Source	Absorption of Photoreceptors (Arbitrary Unit)				
	*σCry* ^1^	*σPhot* ^2^	*σP*_r_^3^	*σP*_fr_^4^	*σP*_r_/*σP*_total_^5^
Standard solar spectrum ^6^	0.500	0.500	0.526	0.474	0.526
Sulfur plasma lamp	0.611	0.613	0.468	0.310	0.601
Red LED (660 nm ^7^)	0.023	0.020	2.277	0.861	0.726
Red LED (630 nm)	0.053	0.046	1.230	0.414	0.748
Green LED (530 nm)	0.085	0.056	0.106	0.033	0.763
Green LED (525 nm)	0.216	0.153	0.162	0.061	0.725
Blue LED (450 nm)	2.935	3.178	0.066	0.154	0.300
Far-red LED (730 nm)	0.133	0.120	0.159	1.236	0.114
White LED (6000 K ^8^)	0.777	0.855	0.464	0.229	0.690
White LED (3000 K)	0.289	0.301	0.774	0.343	0.693
Incandescent lamp	0.082	0.081	0.516	0.640	0.447
Infrared incandescent lamp	0.000	0.000	0.356	0.772	0.315

^1^ Light absorption of cryptochrome; ^2^ Light absorption of phototropin; ^3^ Light absorption of red-absorbing form of phytochrome; ^4^ Light absorption of far-red-absorbing form of phytochrome; ^5^ Absorption ratio between red-absorbing form of phytochrome and total phytochromes; ^6^ Direct and circumsolar spectrum from standard tables of reference for solar spectral irradiance (G173-03; ASTM, 2012); ^7^ Peak emission wavelength; ^8^ Correlated color temperature.

**Table 3:** Photomorphogenic characteristics of cucumber plants grown under each treatment. See Section 2.4 for treatments.

Treatment	Number of Leaves	Total Leaf Area (cm^2^)	Projected Area Ratio (%) ^1^	Petiole Length (cm)	Internode Length (cm)
AS	6.0 ^a^	632.6 ^a,2^	78.3 ^a^	6.6 ^a^	4.1 ^a^
HPS_0_	6.0 ^a^	670.1 ^a^	57.9 ^b^	3.5 ^b^	1.5 ^b^
FL_0_	6.3 ^a^	688.2 ^a^	57.5 ^b^	3.0 ^b^	1.8 ^b^
RB_0_	6.7 ^a^	656.0 ^a^	43.1 ^c^	3.3 ^b^	1.9 ^b^
HPS_m_	6.3 ^a^	709.2 ^a^	77.8 ^a^	7.2 ^a^	4.6 ^a^
FL_m_	5.7 ^a^	758.3 ^a^	79.2 ^a^	6.3 ^a^	4.2 ^a^
RB_m_	7.0 ^a^	712.9 ^a^	71.7 ^a^	6.9 ^a^	4.1 ^a^

^1^ Ratio between leaf area projected to horizontal plane and total leaf area; ^2^ Different letters (a–c) indicate significantly different means according to Fisher’s least significant difference test (*p* < 0.05).

**Table 4:** In situ leaf photosynthetic rate and dry mass of cucumber plants. See Section 2.4 for treatments.

Treatment	In Situ Photosynthetic Rate (μmol_CO__2_ m^−2^ s^−1^)	Dry Mass (g)
AS	2.84 ^d,1^	3.40 ^a,b^
HPS_0_	3.16 ^c^	2.35 ^c^
FL_0_	4.30 ^b^	2.29 ^c^
RB_0_	5.12 ^a^	2.58 ^c^
HPS_m_	2.13 ^e^	3.83 ^a^
FL_m_	2.02 ^e^	2.97 ^b,c^
RB_m_	2.17 ^e^	3.44 ^a,b^

^1^ Different letters (a–e) indicate significantly different means according to Fisher’s least significant difference test (*p* < 0.05).

## Supplementary Materials

The following are available online at https://www.mdpi.com/2223-7747/9/5/556/s1, Table S1: The photon flux density composition of the light source in each treatment.
