# Supplementary material for: Quantification of Spectral Perception of Plants with Light Absorption of Photoreceptors
Source: Plants (Basel). 2020 Apr 27;9(5):556. doi: 10.3390/plants9050556 (PMC7285096; doi:10.3390/plants9050556)
Supplement: Supplementary file 1 [file plants-09-00556-s001.pdf]

plants

Table S1. The photon flux density compositions by of the light source in each treatment. See 2.4 for treatments. See Figures 1b and 2 for the spectra of the light sources and treatments.

Treatment	Photosynthetic Photon Flux Density (μmol m^{-2} s^{-1}, 400–700 nm) (Far-Red Photon Flux Density, μmol m^{-2} s^{-1}, 700–800 nm)									
	Sulphur Plasma Lamp, Green-Cut Filtered [1]	Incande - Scent Lamp	High Pressure Sodium	Fluore-scent Lamp	Light-Emitting Diodes				Infrared Incande-scent Lamp	Total
					Red (660 nm)	Blue (450 nm)	Green (525 nm)	Far-Red (730 nm)		
AS	125.0 (20.4)	75.0 (86.2)	-	-	-	-	-	-	-	200.0 (106.6)
HPS_0	-	-	200.0 (5.9)	-	-	-	-	-	-	200.0 (5.9)
FL_0	-	-	-	200.0 (1.5)	-	-	-	-	-	200.0 (1.5)
RB_0	-	-	-	-	160.0 (2.4)	40.0	-	-	-	200.0 (2.4)
HPS_m	-	-	158.4 (4.6)	-	-	21.8	20.7	(45.5)	-	200.9 (50.1)
RB_m	-	-	-	-	36.2 (0.4)	27.2	136.7	(44.3)	-	200.1 (44.7)
FL_m	-	-	-	169.5 (1.3)	-	11.9	-	-	18.6 (66.1)	200.0 (67.4)

[1] Filtered with green-cut filter (Filter 1581, Gamcolor, Los Angeles, CA, USA).
